# Bilateral Knee Septic Arthritis in a Seven-Month-Old Girl

**DOI:** 10.7759/cureus.37354

**Published:** 2023-04-10

**Authors:** Ahmad N Boeisa, Ali AL Khalaf

**Affiliations:** 1 Pediatric Orthopedic Surgery, Almoosa Specialist Hospital, Al-Ahsa, SAU; 2 College of Medicine, King Faisal University, Al Hofuf, SAU

**Keywords:** pediatric infectious disease, pediatric orthopedic surgery, knee effusion, bilateral knee involvement, septic arthrits

## Abstract

Septic arthritis (SA) is a type of joint inflammation caused by an infection. It is an orthopedic emergency that requires immediate treatment to avoid serious complications such as joint destruction, osteomyelitis, and sepsis. We present a case of bilateral knee SA in a seven-month-old female who presented to our emergency department with left knee SA, followed by right knee SA one month later.

## Introduction

Septic arthritis (SA) is defined as joint inflammation induced by an infectious agent, most commonly a bacterial one but also including fungal, mycobacterial, viral, or other uncommon pathogens. SA is typically monoarticular, affecting a single large joint such as the hip or knee; however, polyarticular SA involving multiple or smaller joints can occur [1]. SA, though uncommon, is an orthopedic emergency that can cause significant joint destruction, increasing morbidity and mortality [2]. Early recognition and prompt treatment are crucial for maintaining joint function [1]. In Western Europe, the incidence of proven SA ranges from four to 10 per 100,000 patient-years per year [3,4]. SA appears to be on the rise, and this rise appears to be attributed to multiple factors that include increased orthopedic-related infections, an increased life expectancy, more medical and surgical interventions being carried out, and increased use of immunosuppressive treatment [5-7]. SA is most prevalent in the elderly and very young children. It is seen more frequently in childhood than at any other age. Individuals with a history of joint disease (such as rheumatoid arthritis, osteoarthritis, crystal arthropathy, and other types of inflammatory arthritis) are more susceptible to SA [2].

## Case presentation

**Figure 1:**
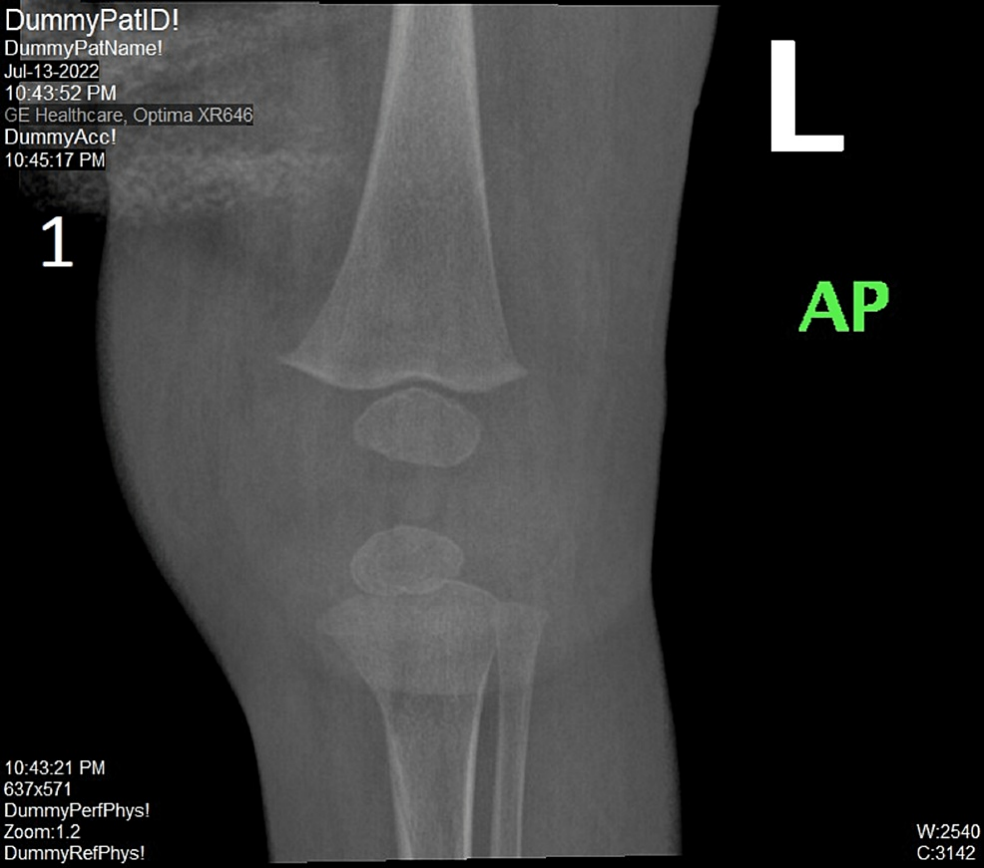
X-ray of the left knee joint AP view This figure shows the expansion of the joint capsule, normal alignment, and no fracture lines. AP: Anterior-posterior.

A seven-month-old girl presented to our emergency department with a complaint of high-grade fever and a refusal to move the left lower limb. She was referred from another hospital as a case of a first-febrile urinary tract infection (*Escherichia coli* sensitive to cefuroxime), for which she was started on cefuroxime. They transferred the patient to rule out malignancy and leukemia. On examination, she was conscious, alert, and hemodynamically stable. She had no skin rashes, oral ulcers, or open wounds. The left knee was hot, tender, and swollen. Laboratory work-up showed leukocytosis (WBC of 26,000 per microliter), thrombocytopenia (platelet count of 966 per microliter), high erythrocyte sedimentation rate (ESR of 115 mm/hour), high C-reactive protein (CRP of 2552 mg/L), lactate dehydrogenase (230 mL/hour), negative blood culture, and a negative Coombs test (antiglobulin testing). She was admitted as a possible case of SA of the left knee joint for further evaluation and management. Initial assessment of the left knee with x-ray showed expansion of the joint capsule (Figure 1).

**Figure 2:**
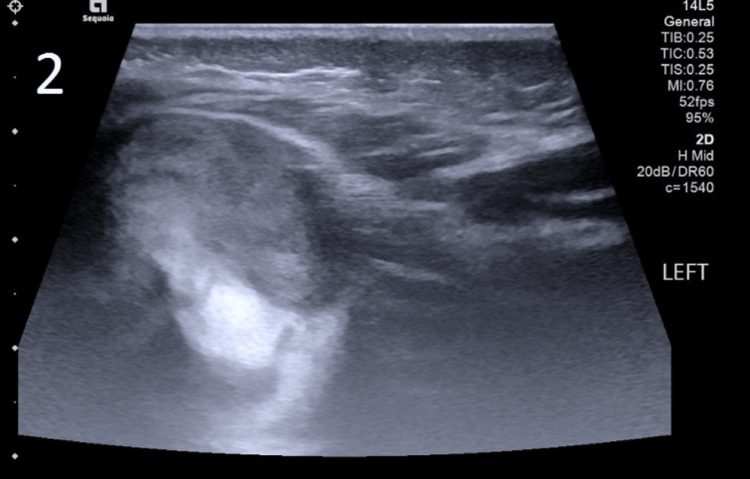
Ultrasound of the left knee joint The ultrasound revealed thickening of the synovium with mild joint effusion.

Knee joint effusion was revealed by ultrasound (Figure 2).

**Figure 3:**
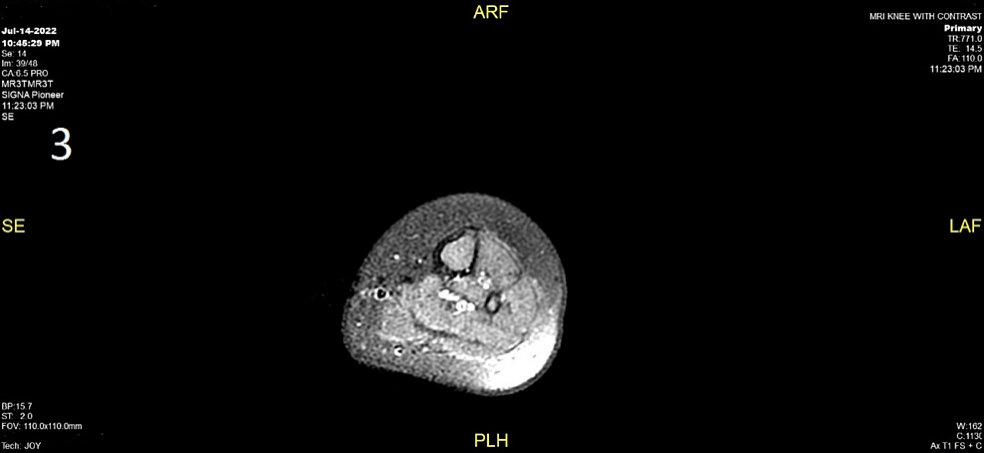
MRI of the left knee joint This figure demonstrates the synovial thickening and enhancement with mild knee joint effusion and surrounding soft tissue edema. The visualized bones show no significant bone marrow edema, periosteal new bone formation, or abnormal enhancement.

MRI of the left knee demonstrated evidence of SA (Figure 3).

**Figure 4:**
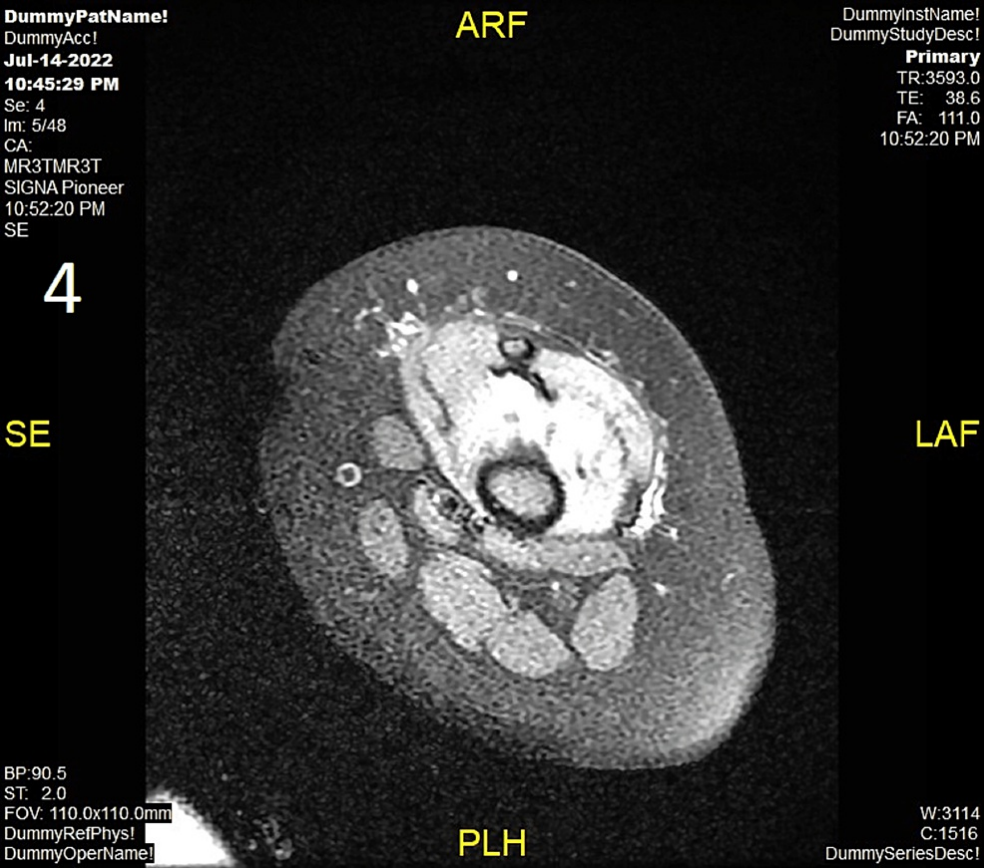
MRI of the right knee joint There is synovial thickening, enhancement, and moderate joint effusion with surrounding soft tissue edema. The visualized bones show no significant bone marrow edema, periosteal new bone formation, or abnormal enhancement.

Left knee arthrotomy was performed, which revealed turbid content, with many WBC and a negative culture. Malignancy was ruled out by bone marrow aspiration. Management included the continuation of IV antibiotics (linezolid and meropenem, to be replaced by teicoplanin and cefipime) as recommended by the pediatric infectious disease team, in addition to methylprednisolone and IV immune globulin (2 g/kg). Significant improvement has been observed immediately following the course of antibiotics, which confirms the diagnosis of knee SA in collaboration with MRI findings. She was discharged in good condition after completing a three-week course of IV antibiotics on analgesia as needed only. One day after stopping steroids, she developed a spike of fever accompanied by swelling and tenderness in her right knee. Lab examination revealed a complete blood count (CBC) (low hemoglobin of 8.6 g/dl, high WBC of 14.3 per microliter, and normal platelet count of 430 per microliter), a high C-reactive protein of 244.70 mg/L, a high erythrocyte sedimentation rate of 87 m/hour, and lactate dehydrogenase of 246 U/I ferritin of 401 micrograms/L. Right knee MRI revealed synovial inflammation and thickening, which was most consistent with SA, and there were no signs of osteomyelitis (Figure 4).

Arthrotomy of the right knee was performed, revealing turbid and thick mucoid fluid, white to yellow in color, a high WBC count of 79,950 cells/ul, a low RBC count of 25 cells/ul, the majority of the cells being neutrophils in large numbers, and a few corticosteroids like crystals. Synovial fluid culture revealed aerobic gram-positive bacilli (identified as *Bacillus subtilis*), confirming the diagnosis of SA. The pediatric infectious disease team advised treatment with an IV antibiotic course for two weeks (composed of meropenem and linezolid). Complete resolution was achieved after the completion of the two-week antibiotic course, and the patient was discharged in good condition. The patient was thriving well before the recent illness, with no reported recurrent infections, or respiratory or gastrointestinal illnesses. She received the national vaccinations up to four months old with no reported complaints. There was no family history of atopy or immunodeficiency. A follow-up plan was established with the patient for further assessment of the underlying reasons for the recurrent SA. Currently, she has returned for several follow-up visits with various specialties in the past nine months with no complaints. She is being evaluated monthly at the pediatric orthopedic clinic, pediatric infectious disease clinic, allergy and autoimmune clinic, and pediatric rheumatology clinic, with no abnormalities detected.

## Discussion

SA of the knee is an inflammatory condition caused by an infectious source. It mainly affects the synovium, followed by most structures found within the joint's borders [3]. SA affects about four to five children out of every 100,000 in Western Europe annually [2,8]. The main causative agent is *Staphylococcus aureus*. *Kingella kingae* is a regional pathogen, which is becoming more common as a causative agent, and respiratory pathogens are especially common in children aged 6-36 months [4]. The hip, knee, and ankle joints are the most commonly affected locations in the body [9]. When compared to other joints, SA of the knee joint is the primary site to be affected in children, accounting for approximately 37%-54.5% of all SA cases in children [8]. Hematogenous spread, direct inoculation, and extension of a contiguous focus of infection are the main routes of infection and the three most frequent underlying mechanisms of SA in children [10]. When the lower limb is affected, acute SA appears as an enlarged, tender joint with a restricted range of motion and fever, and weight-bearing is frequently avoided [8,11]. Immunosuppression or recent antibiotic use may mask the diagnosis of a septic joint [8]. Osteomyelitis, viral arthritides, and juvenile rheumatoid arthritis are all possible differential diagnoses [12].

Several studies have found that the inability to bear weight and a temperature greater than 38.5°C are the most reliable clinical signs distinguishing SA from a transient inflammatory process [11]. Studies have shown that WBC counts greater than 12.0 x 10^9^ cells/L, an ESR greater than 40 mm/hour, and a CRP greater than 20 mg/L are significantly more common in SA when compared to transient synovitis [13]. X-rays should be used to assess the involved joint and adjacent bones to rule out possible etiologies such as fractures, osteomyelitis, or tumors. The most sensitive test for detecting the presence of joint effusion is an ultrasound of the involved joint, which is seen in 91% of patients with SA [8]. MRI is the most precise diagnostic imaging modality, particularly for detecting adjacent osteomyelitis or abscesses [8]. A cell count, Gram stain, microscopy, culture, and sensitivities should be performed as soon as possible.

In some studies, a synovial fluid WBC count of more than 50 x 10^9^ cells/L was used as a cutoff to differentiate between SA and transient synovitis [13]. As previously stated, SA is an orthopedic emergency that is difficult to diagnose. If the disease cannot be ruled out, antibiotic therapy should be started as soon as possible. If SA is strongly suggested, empiric treatment is started after collecting synovial fluid and blood samples. Clindamycin and first-generation cephalosporins are both suitable antibiotics. A two-week course is generally adequate in simple cases [8,14]. If an adjacent bone is affected, antibiotic therapy is then continued for at least three weeks [15]. Dexamethasone can be given to reduce inflammation, which may result in a shorter admission period [16]. To monitor the long-term effects of SA, a two-year follow-up with orthopedic surgery is recommended [8]. The primary goal is monitoring the cartilage damage and growth disruption caused by growth plate damage. Although these complications are uncommon, early detection and treatment are usually associated with a preferable prognosis.

## Conclusions

SA is an emergency condition that can result in permanent joint damage. Early recognition and proper management are crucial for the preservation of joint function. Several risk factors are recognized for predisposition to SA, including open wounds, diabetes, and immunodeficiency. In our patient, no risk factors were found, emphasizing the importance of putting SA at the top of the differential diagnosis in any patient who presents with a swollen knee, even if no risk factors have been identified.
